# The Peroxidase-like Nanocomposites as Hydrogen Peroxide-Sensitive Elements in Cholesterol Oxidase-Based Biosensors for Cholesterol Assay

**DOI:** 10.3390/jfb14060315

**Published:** 2023-06-07

**Authors:** Olha Demkiv, Wojciech Nogala, Nataliya Stasyuk, Nadiya Grynchyshyn, Bohdan Vus, Mykhailo Gonchar

**Affiliations:** 1 Institute of Cell Biology, National Academy of Sciences of Ukraine, 79005 Lviv, Ukraine; olgademkiv81@gmail.com (O.D.); stasuk_natalia@ukr.net (N.S.); 2 Faculty of Veterinary Hygiene, Ecology and Law, Stepan Gzhytskyi National University of Veterinary Medicine and Biotechnologies, 79000 Lviv, Ukraine; ngrynchyshyn@ukr.net; 3 Institute of Physical Chemistry, Polish Academy of Sciences, 01-224 Warsaw, Poland; wnogala@ichf.edu.pl; 4 Department of Electronics and Information Technology, Lviv Polytechnic National University, 79000 Lviv, Ukraine; bogdan.vus@googlemail.com

**Keywords:** cholesterol oxidase, sensor, peroxidase-like nanozymes, platinization, amperometric biosensor, cholesterol assay

## Abstract

Catalytically active nanomaterials, in particular, nanozymes, are promising candidates for applications in biosensors due to their excellent catalytic activity, stability and cost-effective preparation. Nanozymes with peroxidase-like activities are prospective candidates for applications in biosensors. The purpose of the current work is to develop cholesterol oxidase-based amperometric bionanosensors using novel nanocomposites as peroxidase (HRP) mimetics. To select the most electroactive chemosensor on hydrogen peroxide, a wide range of nanomaterials were synthesized and characterized using cyclic voltammetry (CV) and chronoamperometry. Pt NPs were deposited on the surface of a glassy carbon electrode (GCE) in order to improve the conductivity and sensitivity of the nanocomposites. The most HRP-like active bi-metallic CuFe nanoparticles (nCuFe) were placed on a previously nano-platinized electrode, followed by conjugation of cholesterol oxidase (ChOx) in a cross-linking film formed by cysteamine and glutaraldehyde. The constructed nanostructured bioelectrode ChOx/nCuFe/nPt/GCE was characterized by CV and chronoamperometry in the presence of cholesterol. The bionanosensor (ChOx/nCuFe/nPt/GCE) shows a high sensitivity (3960 A·M^−1^·m^−2^) for cholesterol, a wide linear range (2–50 µM) and good storage stability at a low working potential (−0.25 V vs. Ag/AgCl/3 M KCl). The constructed bionanosensor was tested on a real serum sample. A detailed comparative analysis of the bioanalytical characteristics of the developed cholesterol bionanosensor and the known analogs is presented.

## 1. Introduction

Cholesterol (cholest-5-en-3β-ol, CHOL) is a crucial lipid molecule that fulfills different biological functions. It is a bile acid precursor, promoting the synthesis of steroid hormones and some vitamins [1]. It plays a crucial role in maintaining the rigidity of cellular membranes and fluidity [2]. CHOL is a major component of the human brain and is necessary for signal transmission [1,2]. The increased cholesterol level in the blood serves as an important biomarker of cardiovascular disease [3]. According to the World Health Organization, an increase in CHOL levels over 6.2 mM resulted in millions of human deaths worldwide and shortened life expectancy by disability-adjusted years. It raises the mortal chance of several illnesses including diabetes, heart disease, lipid metabolism issues, cerebral thrombosis, brain thrombosis, arteriosclerosis and hypertension [3,4]. This is due to the possibility of CHOL accumulating in blood vessel walls and creating plaque, which can restrict blood flow and raise the risk. Therefore, CHOL assays are crucial for medical diagnostics and, respectively, global healthcare.

Different approaches including colorimetric [5,6], fluorometric [7,8], high-performance liquid chromatography (HPLC) [9,10,11] and gas chromatography-mass spectrometry [12] have been used to determine CHOL. However, most of these approaches are highly sophisticated, time-consuming and require a special technique for routine use. In recent years, the immunoassay method for CHOL detection is widely used in clinical laboratories due to its high sensitivity and specificity [13]. This is advantageous in clinical laboratories where efficiency and productivity are essential. Despite the immunoassay techniques of CHOL testing having their advantages, conventional methods such as biosensors approaches do not require specialized reagents and equipment [14]. Therefore, the development of biosensors for CHOL assay is of great interest. 

Electrochemical sensors are one of the leading techniques for detecting many analytes in biological fluids with high accuracy. For the CHOL assay, highly selective enzymatic electrochemical sensors based on the high substrate-specific cholesterol oxidase (ChOx) and peroxidase (HRP) [15] or in combination with various nanomaterials [14,16] have been created.

ChOx is a FAD-containing enzyme which catalyzes the oxidation of CHOL to cholest-4-en-3-one with the reduction of oxygen to hydrogen peroxide (H_2_O_2_) [17]. The detection of H_2_O_2_ could be an indirect quantification of CHOL levels. The level of CHOL can be determined by detecting the current produced from the transformation of the H_2_O_2_ generated during the enzymatic reaction of ChOx. However, this approach of indirect detection of CHOL requires a high applied working potential and, as a result, may lead to insufficient selectivity due to the impact of other interfering compounds in real samples [18,19,20]. To decrease the value of the applied working potential, there are generally used electron mediators such as Prussian Blue [21] or hydroxymethylferrocene [22]. Recently, amperometric biosensors for CHOL analysis based on direct electron transfer (DET) between ChOx and the electrode working at low potentials have been reported [14,19,20]. It is known that nanoparticles can provide new possibilities for the construction of biosensors with excellent analytical properties due to their unique properties such as excellent conductivity, high porosity and a large surface-to-volume ratio [14,16,17,18,19,20,21,22,23]. It has been constructed ChOx-based biosensors where the Chox was immobilized on the surface of nanomaterials, namely, carbon nanotubes (CNTs) [24], graphene [25], metallic nanoparticles (NPs) [26,27,28,29,30], transition metals nanomaterials [30,31], rare earth metal oxides nanomaterials [26,30], conductive polymers [23,32,33], quantum dots [34], hybrid combinations of them [29,35] and even with redox mediators (K_4_[Fe(CN)_6_], thionine, etc.) [19,21,26]. CNTs exhibit many attractive properties. However, the adsorption of small amounts of chemicals can lead to a dramatic change in CNT conductivity. To improve the properties of carbon materials, in particular, sensitivity, they should be functionalized with metal NPs such as Au and Pt [27,31,33,36,37]. Au NPs are often widely used in electrochemistry due to their ease of synthesis and chemical stability [36,37,38,39,40,41,42,43].

To construct CHOL biosensors, different organic polymers in combinations with NPs have been also used, namely, AuNPs/CNTs/poly(allylamine hydrochloride) [44], polyaniline [45], AuNPs/Poly-(diallyldimethyl-ammonium chloride)/CNTs, polypyrrole/AuNPs/CNTs [45,46], PANi/CNC/IL/GLU, 4-(4H-dithienol[3,2-b: 2′,3′-d]pyrrole-4)aniline polymer [45] or poly(N[3-(trimethoxysilyl)propyl]aniline] [47]. All proposed bioelectrodes facilitate DET to the electrode.

Among the metal NPs, Pt-Pd [29], Cu-Pt-Bi [35], AgNPs [38], PdNPs [39], CeO_2_-NR [40,48], AuNPs [28,35,38,39,44,46,47,48,49,50], TiO_2_ [16,26,29] and Cu_2_ONPs [26], hybrid nanocomposites are often used in biosensors for cholesterol detection. 

Unlike natural enzymes, which are often unstable and can be easily denatured under harsh conditions, nanozymes (NZs) are highly stable and can maintain their catalytic activity under a wide range of conditions. It is known that NZs as stable cost-effective mimics of natural enzymes may be promising catalysts in food and environmental biotechnology, biosensorics, alternative energy and medicine. Enzyme-like NZs, including metallic nanocomposites, are promising catalysts for biosensing applications [51,52,53,54]. Artificial peroxidase being able to utilize as a substrate hydrogen peroxide, the final product of oxidase-catalyzed splitting of different practically important analytes, may be promising hydrogen peroxide-selective chemosensing membrane, coupled with a relevant oxidase in the biosensing layer. Recently, it has been proposed using a Prussian blue (PB) mediator that encourages electron transfer and plays a role as an ‘‘artificial peroxidase” that catalyzes the H_2_O_2_ reduction at low potential values [55]. Its low cost and selectivity to H_2_O_2_ reduction make PB promising candidates for the construction of biosensors.

In the current paper, we report the construction of an amperometric “bionanosensor” based on using natural ChOx and several new nanocomposites with HRP-like catalytic activity. The best prototype of such as a bionanosensor with an architecture of a bioselective layer as GhOx/nCuFe/Pt, located on a glassy carbon electrode (GCE), for CHOL assay on the model of human serum. The developed bionanosensor due to its sensitivity, validity, low cost and high selectivity will be prospective for application in clinical laboratories for CHOL assay in blood serum.

## 2. Materials and Methods

### 2.1. Reagents

All reagents and solvents used in this work were of analytical grade from Sigma–Aldrich (Steinheim, Germany). Cholesterol oxidase (ChOx), dipotassium hydrogen phosphate, potassium dihydrogen phosphate, cholesterol (CHOL), ascorbic acid (AA), uric acid (UA), salicylic acid (SA), glutathione (GN), glucose (GU), chloroplatinic (IV) acid, creatinine (CN), copper(II) sulfate, hydrogen tetrachloroaurate(III) trihydrate, cerium(III) chloride, o-dianisidine, hydrogen peroxide (30%), glutaraldehyde (25%), cetyltrimethylammonium bromide (CTAB), cysteamine, isopropanol (98%) and Triton X-100 were from Sigma–Aldrich.

### 2.2. Synthesis of HRP-like NZs

All NPs were obtained by the chemical reduction method [56,57]. nCeAu were obtained by the following procedure: 5 mL 10 mM HAuCl4 and 5 mL 10 mM Ce(HCO_3_)_4_ were mixed with 0.1 mL 10 mM CTAB and 1.5 mL 50 mM ascorbic acid. The mixture was stirred for 10 min at 20 °C. The scheme of the synthesis of nAgCu was as follows: 2 mL 5 mM AgNO_3_ and 5 mL 1 mM CuSO_4_ were mixed with 0.1 mL 10 mM CTAB and 1 mL 100 mM ascorbic acid.

nCuFe were synthesized according to the following procedure: 4 mL 1 mM CuSO_4_ and 2 mL 10 mM FeCl_3_ were mixed with the following addition of mixture containing 0.1 mL 10 mM CTAB and 0.1 mL 20 mM NaBH_4_. The mixture was stirred for 15 min at 20 °C and centrifuged at 8000× *g*. All obtained NPs were dried at 80 °C for 24 h. The total concentration of NPs was determined gravimetrically. 

The morphology and sizes of synthesized NPs were studied by the use of a scanning electron microscope (SEM) FEI Nova NanoSEM 450.

### 2.3. Evaluation of HRP-like Activity of the Synthesized NZs 

To determine the HRP-like activity of the synthesized NZs in solution, the spectrophotometric method with o-dianisidine was applied [55]. The specific activity (U/mg) was calculated according to the formula [56]:

$$Specific\;activity=\frac{D_{525}\cdot V_{t}\cdot N}{13.38\cdot V_{NZ}\cdot c\cdot t},$$

where

*D*_525_—optical density of the test tube; 

*N*—dilution of the tested sample before injection in reaction mixture; 

*V_t_*—total volume of reagents in test tube, mL;

13.38—millimolar extinction coefficient of the coloured product, mM^−1^∙cm^−1^;

*V_NZ_*—aliquot of added NZs, mL;

*c*—initial concentration of NZs, mg·mL^−1^;

*t*—time, min.

### 2.4. Construction of Amperometric Biosensors 

The constructed sensors were characterized using amperometry in a three-electrode configuration with an Ag/AgCl/KCl (3 M) reference electrode, a Pt-wire counter electrode and a working GCE [56]. 

### 2.5. Study of H_2_O_2_-Sensing Ability of the Electrodes, Modified with Peroxidase-like NPs

The electrochemical properties of synthesized NZs were studied by CV and chronoamperometry and the profiles of amperometric outputs were compared. The most electroactive toward H_2_O_2_ NPs were taken for the construction of biosensor on CHOL.

### 2.6. Modification of GCE with nPt

Platinum NPs (nPt) were synthesized by the method of electrodeposition under potentiodynamic mode on a surface of GCE from the mixture containing 6 mM H_2_PtCl_6_ in 0.5 M sulfuric acid in the range from −0.5 to +1.00 V at 25 mV·min^−1^ during 6 cycles vs. Ag/AgCl/3 M KCl. The obtained platinized GCE was rinsed with 50 mM PB, pH 7.5 and kept till usage. 

### 2.7. Construction and Characterization of Bionanoelectrodes

To construct nanostructured bioelectrodes, ChOx was immobilized as a film on HRP-like NZs and immobilized on GCE surface (intact or platinized (nPt/GCE) variants).

To immobilize NZs, an aliquot of NZs (0.002 mL, 0.2 mg/mL) was dropped on the GCE or nPt/GCE electrodes and dried at RT. Then, the aliquot of a mixture (0.002 mL) containing cysteamine (1 mM) and glutaraldehyde (1%) was added to the NPs layer and incubated up to film formation. Finally, the functionalized electrodes NZ/GCE or NZ/nPt/GCE were rinsed with 50 mM PB, pH 7.0 and used for ChOx immobilization using cross-linking reaction. For this purpose, an aliquot of ChOx solution (0.005 mL, 15 U·mL-1) was placed onto the NZ-modified electrode and dried. The fabricated bionanoelectrodes were stored at +4 °C until use. 

### 2.8. Analysis of CHOL in Serum

As a standard CHOL solution, it was used 12.95 mM CHOL in 50 mM PBS, pH 7.5, containing 1% Triton-X100 and 20% isopropanol. The sample of serum was tested from a healthy volunteer (woman, 35 years old). Reference CHOL assay was performed using an enzymatic analytical kit “Chromocholesterol” [57].

### 2.9. Statistical Analysis

All experiments were carried out three times (n = 3) and measurements were performed in two parallels. The statistical parameters and all figures were calculated and built using Origin 8.5 Pro. 

Sensitivity (A·M^−1^·m^−2^) was calculated as follows: Sensitivity = B/S, where B—the slope for the dependence of current on analyte concentration in linear range (A·M^−1^); S—the surface area of the working electrode (m^2^). 

The limit of detection (LOD) was calculated by using the standard deviation (SD) of the blank current signals and the B value according to the formula: LOD = (3∗ SD/B).

## 3. Results and Discussion

### 3.1. Synthesis and Characterization of the Nanocomposites Most Sensitive to Hydrogen Peroxide

The aim of the current research was to construct novel NZs-based biosensors coupled with ChOx, promising for medical diagnostics with the usage of NZs as peroxidase mimetic catalysts. Thus, the obtained NPs were tested on their pseudo-peroxidase activity in the reaction with o-dianisidine. It was demonstrated that several NPs, especially nCeAu, nAuHCF, nCuFe and nCoHCF possessed the highest HRP-like activities among all others (Table S1). The most active in solution NZs, namely, nCeAu, nAuHCF, nCuFe and nCoHCF were taken for morphological characterization by SEM and X-ray microanalysis. SEM and XRM provided information on the size and shape of the studied samples (see Supplementary Materials, Figure S1). It was shown that the synthesized NZs, in particular, nCuFe and nCeAu have a shape close to spherical with a diameter of less than 30 nm. The XRM images (nCuFe and nCeAu) demonstrate the peaks for metals of the composites. The morphology of nAuHCF was presented in our earlier paper [56].

The obtained catalytically active NZs with a high activity in solution, namely, nCeAu, nAuHCF and nCuFe were chosen for further investigation.

**Figure 1:**
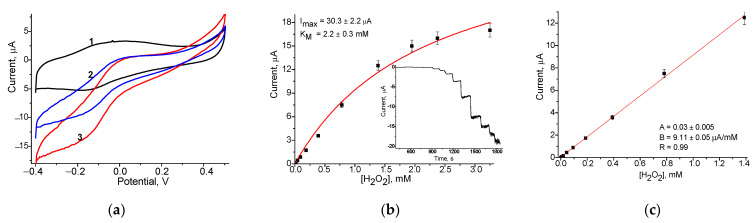

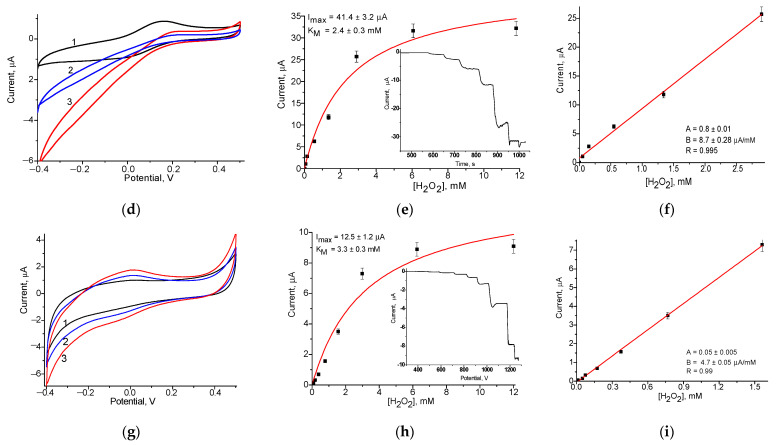
Amperometric characteristics of nCuFe/GCE, nAuHCF/GCE and nCeAu/GCE: (**a**,**d**,**g**)—CV under increased concentrations of H_2_O_2_: (1)—0 mM; (2)—2.5 mM; (3)—5.0 mM at 20 mV·s^−1^; (**b**,**e**,**h**)—dependences of current outputs on the concentration of H_2_O_2_ and chronoamperometric current responses (the insert) upon H_2_O_2_; (**c**,**f**,**i**)—calibration graphs for subsequent additions of H_2_O_2_ (an electrode area of 7.06 mm^2^).

To evaluate the possibility of using the synthesized NZs as peroxidase-like elements, sensitive to H_2_O_2_ generated in ChOx-based biosensor, the GCE electrodes, modified with different NZs (nCeAu, nAuHCF and nCuFe) were tested in model experiments with H_2_O_2_ as an analyte. The amperometric characteristics of several H_2_O_2_-sensitive chemosensors are presented in Figure 1. The comparison was made in relation to the biosensor analog, for which, instead of artificial “nanoperoxidase”, natural horseradish peroxidase (HRP) was exploited. The electrochemical behavior of NZs, immobilized on the surface of GCEs, was screened by CV and chronoamperometry (Figure 1). Cyclic voltammograms (Figure 1a) for all types of NZs-based chemoelectrodes demonstrated that a strong reduction peak caused by H_2_O_2_ decomposition occurred at potentials between −100 mV to −300 mV (vs. Ag/AgCl). For further experiments, including chronoamperometry, the potential of −200 mV was taken as the optimal potential. The amperometric characteristics of several H_2_O_2_-sensitive chemosensors are presented in Figure 1. The results of chronoamperometry analysis and correspondent calibration graphs at −200 mV are presented in Figure 1b,c, respectively. Thus, the obtained HRP-like NZs, in particular, nCeAu, nAuHCF and nCuFe are prospective artificial HRPs for the development of cholesterol oxidase-based biosensors.

The analytical characteristics of the developed chemosensor electrodes in comparison with the HRP-based bioelectrode under injected H_2_O_2_ are presented in Table 1. The linear ranges, limits of detection (LOD), I_max_ values (for an electrode area of 7.06 mm^2^) and sensitivities were calculated.

As shown, under reductive voltage (−200 mV), the nAuHCF/GCE and nCuFe/GCE have the highest sensitivity (1290 and 1232 A∙M^−1∙^m^−2^, respectively) and they were used in conjunction with cholesterol oxidase for further development of an amperometric biosensor for cholesterol assay.

### 3.2. Platinization of GCE for Construction of H_2_O_2_- and Cholesterol-Sensitive Sensors

**Figure 2:**
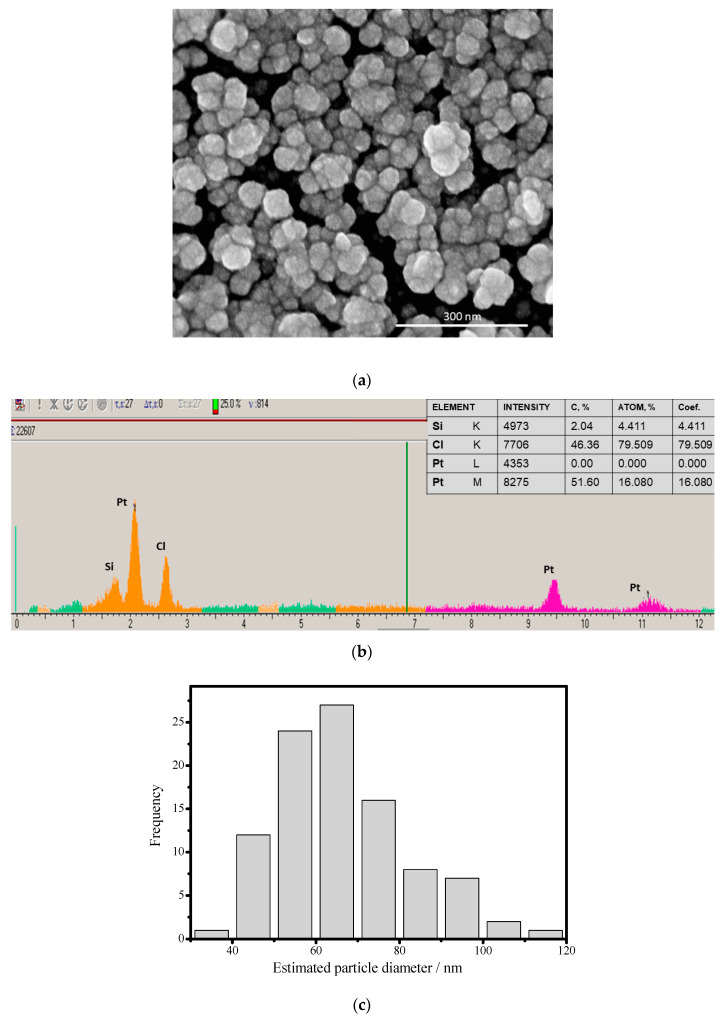
Morphological properties of electrodeposited Pt NPs formed on the surface of GCE: (**a**) SEM image, (**b**) X-ray spectral microanalysis of nPt and (**c**) the size distribution for the Pt particles electrodeposited on GCE.

The literature shows that the additional modification of the electrode surface with platinum leads to improved electroconductivity and catalytic properties of the modified electrodes [58,59,60,61,62,63]. For modification of the GCE surface, we have used the approach of electrodeposition of platinum in potentiodynamic mode. Morphological and structural properties of the electrodeposited Pt NPs were studied, including SEM micrographs and X-ray spectrograms (Figure 2). The distribution (Figure 2c) calculated from the SEM (Figure 2a) confirms that the formed Pt layer is nanosized with medium size of crystallites near 62 nm. The data of XRM of the Pt NPs demonstrates the characteristic peaks for Pt^0^, proving the generation of nPt (Figure 2b).

**Figure 3:**
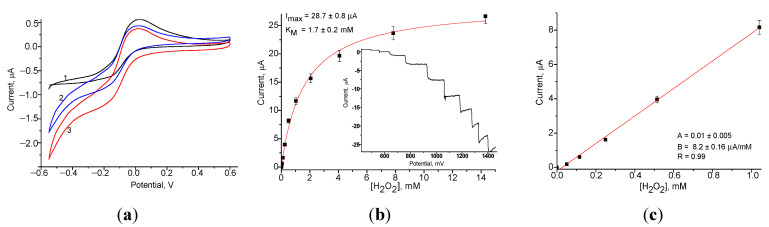
Analytical characteristics of the chemosensor nPt/GCE: (**a**) CV responses at various H_2_O_2_ concentrations: (1)—0 mM, (2)—2.5 mM and (3)—5.0 mM; (**b**,**c**) chronoamperograms (inserted) and calibration curves for H_2_O_2_ determination at working potential −200 mV (an electrode area of 7.06 mm^2^).

The electrochemical properties of nPt/GCE as a chemosensor for H_2_O_2_ were investigated (Figure 3). 

The CV (Figure 3a) demonstrates the reduction peak that occurs at a potential between −100 mV and −300 mV under injected 0.05–5 mM H_2_O_2_ solutions, respectively. The chronamperometric outputs and calibration graph for H_2_O_2_ assay at −200 mV are shown in Figure 3 (b and c, respectively). The maximum current response of the nPt/GCE was 28.7 ± 0.8 µA (electrode surface is 7.06 mm^2^) with the apparent Michaelis–Menten constant (K_M_^app^)—1.7 ± 0.2 mM.

### 3.3. Construction and Analytical Properties of ChOx/NZs-Based Bionanosensors

#### 3.3.1. Construction of Unmodified ChOx/NZs-Based Bionanosensors

**Figure 4:**
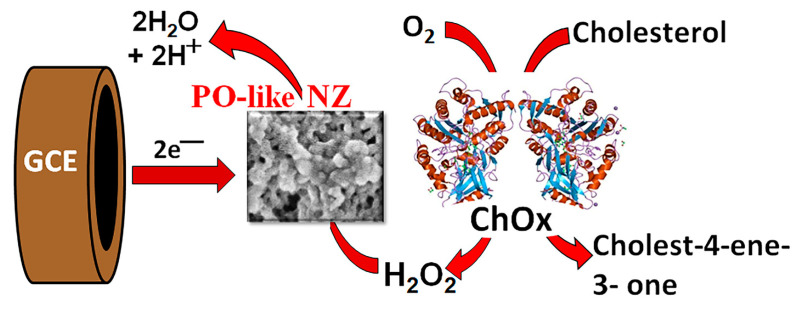
Principal scheme of the functionality of the ChOx-based biosensor.

Selective to hydrogen peroxide HRP-like NZs, coupled with cholesterol oxidase, were taken for the development of amperometric biosensors on CHOL. The main principle of the biosensor functioning is based on the direct detection of H_2_O_2_, produced as a result of CHOL oxidation under ChOx digestion (Figure 4).

**Figure 5:**
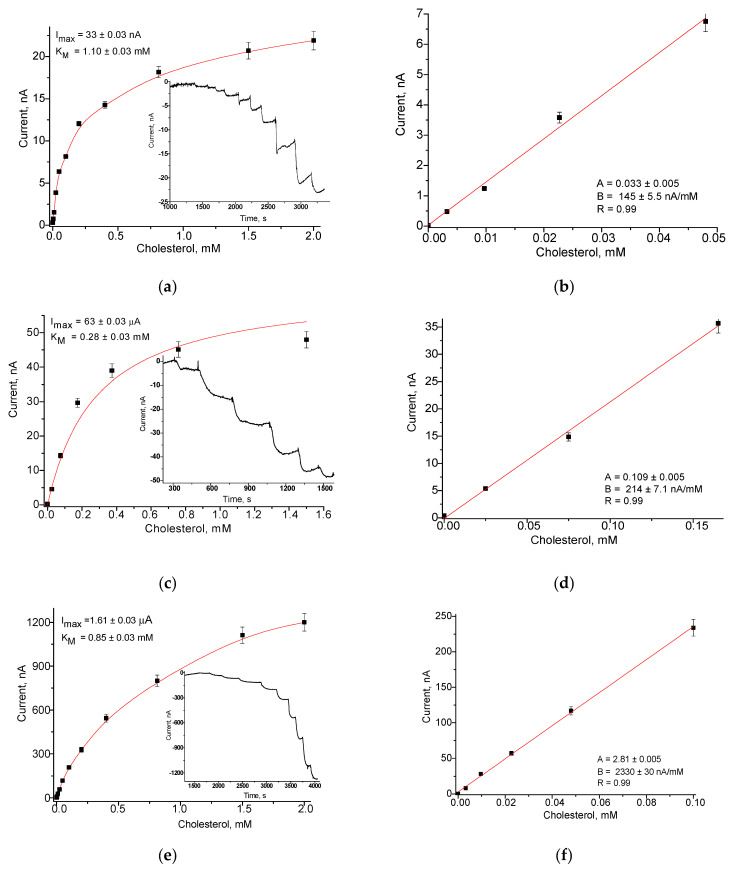

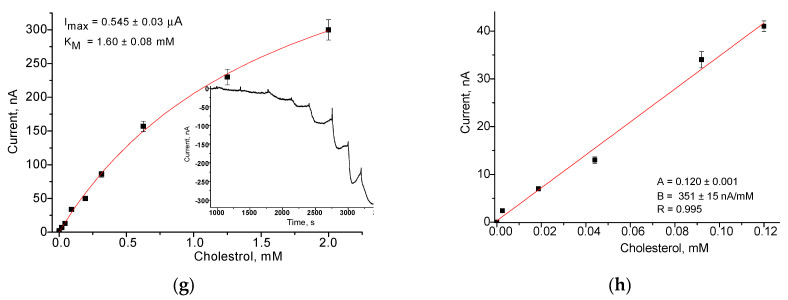
Bioanalytical characteristics of the constructed bionanosensors GhOx/nCeAu/GCE (**a**,**b**), GhOx/nAuHCF/GCE (**c**,**d**), GhOx/nCuFe/GCE (**e**,**f**) and GhOx/HRP/GCE (**g**,**h**) at −250 mV: chronamperometric current responses (**a**,**c**,**e**) and corresponding calibration curves (**b**,**d**,**f**) upon additions of CHOL (an electrode area of 7.06 mm^2^).

The calibration curves for the developed bionanosensors and the corresponding chronamperograms (inserts) are shown in Figure 5. The main bioanalytical characteristics of constructed biosensors are shown in Table S2.

The comparison of the constructed nanostructured bioelectrode with architecture ChOx/nCuFe/GCE with the analog based on natural peroxidase (GhOx/HRP/GCE) shows a significant increase (6.55-fold) in sensitivity (49 A·M^−1^·m^−2^ and 331 A·M^−1^·m^−2^, respectively). Possibly, it can be explained by a higher electron transfer rate between artificial nanoperoxidases and the electrode surface. 

#### 3.3.2. Construction and Bioanalytical Properties of Bionanosensors Based on ChOx and nCuFe Using Platinized GCE

**Figure 6:**
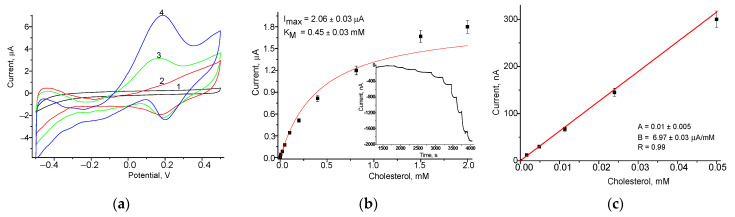
Analytical characteristics of the bionanosensor GhOx/nCuFe/nPt/GCE: (**a**) CV responses at various CHOL concentrations: (1)—0 mM, (2)—1.5 mM, (3)—3.0 mM, (4) —4.5 mM; (**b**,**c**) chronoamperograms (inserted) and calibration curves for CHOL determination at working potential–250 mV (an electrode area of 1.76 mm^2^).

It is known that platinized electrodes by NPs sufficiently increase their electroactivity [58], so we have studied a possible effect of “nanoplatinization” on the working GCE bioanalytical parameters of the derived bionanosensor ChOx/nCuFe/nPt/GCE. To choose the optimal working potential, CV was applied (Figure 6a). The CV profiles of the sensors were compared with GCE in the present and in the absence of CHOL in the electrochemical cell (Figure 6). An amperometric response on CHOL was tested for CHOL concentration from 5 µM to 5 mM. The linearity of the proposed biosensor is in the range between 5 µM and 50 µM. The maximal detected signal was 2060 ± 30 nA with K_M_^app^ for CHOL 0.45 ± 0.03 mM (Figure 6b). The sensitivity within the linear range was estimated as 3960 ± 35 M^−1^⋅m^−2^ (for electrode with a working area of 1.76 mm^2^), LOD was 0.2 µM, and a response time was 10 s.

As shown, the bionanosensor GhOx/nCuFe/nPt/GCE possesses the highest sensitivity to CHOL (3960 A·M^−1^·m^−2^) is explained by amplification catalytic effects of nanocomposites, namely, nCuFe and nPt, which cover the GCE. The proposed bionanosensor has a 12-fold higher sensitivity compared to GhOx/nCuFe/GCE without nPt-layer (331 A·M^−1^·m^−2^) (Figure 6). The Michaelis–Menten constant (K_M_^app^) for the platinized nanostructured bioelectrodes is much lower, indicating the better substrate affinity of the sensing layer to cholesterol. 

The sensitivity of the ChOx/nCuFe/nPt/GCE sensor has been compared with the literature data, as shown in Table 2. To date, a variety of materials such as CNTs, polymers, metal NPs and graphene are used for the construction of biosensors for CHOL detection [24,26,37,46,48,49,50,51,52,53,54,55,56,57,58,59,60,61,62,63]. The developed bionanosensor ChOx/nCuFe/nPt/GCE has several advantages such as lower cost, high sensitivity and selectivity. Although, recently, there was described ChOx-based biosensor using MoS_2_-Au NZ with much better sensitivity (4460 μA mM^−1^ cm^−2^); however, high sensitivity is not always preferable, first of all, for metabolites which are present in human biological liquids in the mM range (glucose, lactate, cholesterol and others) because the very high sensitivity resulted in the necessity to dilute the tested samples before analysis.

It should be mentioned that most of the described biosensors exploit electron transfer mediators or operate at high potentials (+0.6 V) which can induce interference and reduce the accuracy of the measurements. So, the sensors developed in the current paper reveal good sensitivity, do not require free-diffusion mediators, have a fast response to the target analyte and the preparation of nanozymes is a simple and cost-effective procedure which is a great advantage compared to the described sensors.

### 3.4. Analytical Properties of the Constructed Bionanosensor

**Figure 7:**
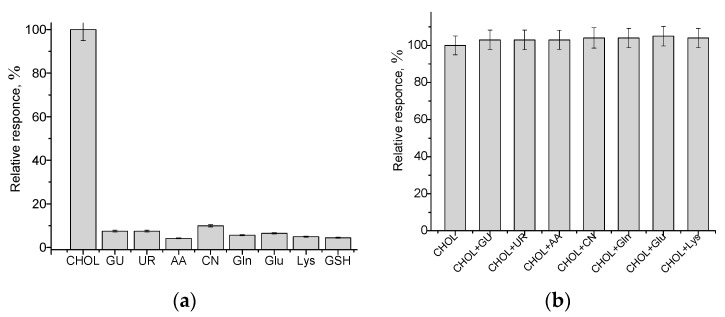
The investigation of the selectivity (**a**) and possible interference effect (**b**) of constructed ChOx/nCuFe/nPt/GCE at various compounds without and with the addition of 0.5 mM CHOL. Glucose (GU), urea (UR), ascorbic acid (AA), creatinine (CN) glutamine (Gln) glutamic acid (Glu), glutathione (*GSH*), lysine (Lys) and cholesterol (CHOL). The analytical signal under the working potential—250 V is presented in relative units (%) related to the maximal current signal on CHOL (100%).

The test on the selectivity of the developed ChOx/nCuFe/nPt/GCE sensor was evaluated by analyzing responses on compounds, usually presented in biological liquids, namely, glucose (GU), urea (UR), ascorbic acid (AA), creatinine (CN), glutamine (Gln), glutamic acid (Glu), glutathione (GSH) and lysine (Lys) (Figure 7). The tested species were added individually in 0.5 mM concentration and amperometric outputs were evaluated. Only a low current response was observed for GU—8%, UR—8%, AA—4.2%, CN—10%, Gln—5.7%, Glu—7% and Lys—5% selectivity graph (Figure 7a).

The indicated compounds were also tested for their possible interfering effect in a mixture with CHOL (Figure 7b). The data clearly demonstrate a very small effect of the tested species on CHOL assay. 

The reproducibility of the constructed biosensor was investigated by analyzing 0.50 mM CHOL seven times for one day. The relative standard deviation (RSD) is shown to be 4.3%, which is a permissible error for clinical analysis. The constructed bionanosensor ChOx/nCuFe/nPt/GCE was checked to run seven consecutive measurements in the 1.0 mM CHOL solution. The obtained sensor revealed an R.S.D. value of 1.8%, indicating good repeatability.

The responses of the independently prepared nanostructured bioelectrodes were tested for 0.5 mM CHOL using chronoamperometry to estimate the repeatability. The relative standard deviation was shown to be 2.7%.

Storage stability of ChOx/nCuFe/nPt/GCE sensor was also investigated by everyday monitoring of its response to 0.5 mM CHOL. The sensor retained 82% of the initial current after seven days of storage of the bionanoelectrode at room temperature, with a slight decrease in activity, indicating storage stability of the electrode.

To show the practical application of the developed bionanosensor, CHOL content was tested in human serum samples (Figure S2). The estimation of CHOL in a real serum sample was provided for two variants of the bionanoelectrode with and without nPt electrodeposition. The biosensors’ data were compared with the values enzymatic kit “Chromocholesterol” (Ukraine) (Table 3).

The results of tests on the accuracy of the developed bionanosensors for the determination of CHOL are shown to meet requirements for the permissible limits of 5.0%. The obtained results approve the possibility of application of the constructed amperometric bionanosensors for CHOL assay in human biological liquids. 

To date, intensive research has been carried out to develop oxidase-based biosensors in combination with catalytic nanomaterials for the assay of natural metabolites—markers of human diseases or indicators of food quality. Although many peroxidase-like NZs had been proposed, some problems still need to be solved, in particular, finding out the mechanism of interaction of peroxidase-like NZs with hydrogen peroxide generated in the enzymatic reaction, and, secondly, the need in optimization of architecture structure of the sensing layer, improvement of storage stability of the enzyme–nanozyme electrodes and protocol settings for practical using such sensors for routine analyses. Moreover, many publications show that the additional modification of the working electrode surface with the metal nanoparticles resulted in the improvement of electroconductivity and catalytic properties of the modified electrodes. In our opinion, the application of peroxidase-like NZs coupled with nanoplatinum layer provided a good platform for the construction of highly sensitive oxidase-based biosensors, suitable for future application in clinical practice including for cholesterol assay. 

## 4. Conclusions

The novelty of the presented work is related to the synthesis of new peroxidase-like metal-based nanocomposites and the evaluation of their functionality as “chemonanosensors” on hydrogen peroxide and as the sensing components in CHOL-based “bionanosensors” for the CHOL assay. The used bi-metallic CuFe nanoparticles were shown to have the best catalytic activity in the sensor’s layer, particularly when using nanoplatinized glassy carbon electrodes. The other advantage of the presented experimental approach for selecting the best peroxidase-like NZs is a combination of testing their intrinsic peroxidase activity in a solution (without applying electric potential) and monitoring their H_2_O_2_-sensing ability on the electrode. Such preliminary experiments make the construction of oxidase-based sensors more reasonable.

The constructed ChOx/nCuFe/nPt/GCE bionanosensor exhibits a high sensitivity (3960 A·M^−1^·m^−2^) for the target analyte, a broad linear range (2–50 µM), satisfactory storage stability and excellent selectivity. The substitution of HRP by nCuFe as HRP-nanozyme in the sensing layer and nanoplatinization of the working electrode resulted in a 12-fold sensitivity increase compared to the non-platinized GCE one. The practical applicability of the constructed bionanosensor was demonstrated by CHOL assay in a human serum sample. Because a high sensitivity of the sensor is not preferable for routine serum analysis (dilution is required), in the future, we plan to control sensitivity by using semi-permeable membranes, covering the outer surface of the bionanoelectrode and testing of such bionanosensor for assay CHOL in a massive lot of clinical samples.

## Figures and Tables

**Table 1:** Analytical parameters of the constructed chemosensors for H_2_O_2._

N	H_2_O_2_-Selective Layer	Sensitivity, A·M^−1^·m^−2^	Linear Range, up to mM	I*_max_*, µA	K_M_^app^, mM
1	nAuHCF/GCE	1232 ± 40	0.02–3.0	41.4 ± 3.2	2.4 ± 0.3
2	nCuFe/GCE	1290 ± 7.1	0.02–1.4	30.3 ± 2.2	2.2 ± 0.3
3	nCeAu/GCE	666 ± 7.0	0.02–1.6	12.5 ± 1.2	3.3 ± 0.3
4	HRP/GCE	351 ± 20	0.05–0.2	4.8 ± 0.2	5.9 ± 1.1

**Table 2:** Comparison of analytical properties of the current NZ-based CHOL bionanosensors with known analogs.

Biosensors	Sensitivity, A·M^−1^·m^−2^	LOD µM	Linear Range, mM	K_M_^app^, mM	Potential, V	Redox Agent	Reference
GlOx/nCeAu/GCE	33 ± 2	4	0.05	1.1	−0.25	nCeAu NZs	This work
GlOx/nAuHCF/GCE	63 ± 4	4	0.18	0.2	−0.25	nAuHCF NZs	This work
ChOx/nCuFe/GCE	321 ± 12	2.8	0.003–0.1	0.85	−0.25	nCuFe NZs	This work
GlOx/nPt/GCE	305 ± 14	1.7	0.02–0.4	2.46	−0.25	nPt NZs	This work
ChOx/nCuFe/nPt/GCE	3960 ± 20	0.2	0.002–0.05	0.41	−0.25	nCuFe/nPt NZs	This work
Ti/NPAu/ChOx–HRP–ChE	29.33	0.01	0.97–7.80	0.64	-	HRP	[60]
Au-f-MWCNT-PPy-ChOx/GCE	101.2	-	2–8	1.66	0.6	K_4_[Fe(CN)_6_]	[37]
ChOx/Graphene/PVP/PANI	347.7	-	0.05–0.10	-	0.6	PANI	[45]
SPE/PANi/CNC/IL/GLU/ChOx	351.9	-	1–12	-	0.6	PANI	[46]
ChOx/CS/ZnO@ZnS/GCE	526.7	20	0.4–3	-	0.3	K_4_[Fe(CN)_6_]	[19]
ChOx/TH/Cu_2_O/GCE	702	0.0018	0.01–1	0.025	-	Thionine	[26]
ChOx/Fe_3_O_4_@PAMAM	739	-	0.1–1.5	-	0.6	K_4_[Fe(CN)_6_]	[64]
ChOx/Fe_3_O_4_@APTES	1019	-	0.1–1	-	0.6	K_4_[Fe(CN)_6_]	[64]
ChOx/AuNP/carbon IDE	1203	24.6	1–10	-	0.6	K_4_[Fe(CN)_6_]	[62]
Pt-NC/enzyme/Nafion	1320	2	0.02–0.48	-	0.6	Pt-NC	[63]
ChOx/MoS_2_-AuNP/GCE	44,600 (4460 μA mM^−1^ cm^−2^)	0.026	0.05–0.048	0.325	-	MoS_2_-AuNPs	[56]
SPE-PEDOT-ChOx-nafion	1.34 μA·mM^−1^	-	0.05–0.8	0.24	–0.51	PEDOT	[51]
ChOx/KMWNTs/GCE	2 μA·mM^−1^	-	0.005–0.016	-	0.6	KMWNTs	[65]
ChOx/CeO_2_-NR	858	680	1–6.5	0.68	1.25	CeO_2_	[48]
Ag/ChOx/ChE/Au NPs/SPE	336	0.009	0.01–15	-	-	Ag	[66,67]

ChOx—cholesterol oxidase; HRP—horseradish peroxidase; ChE—cholesterol esterase; TH–thionine; APTES—3-aminopropyltriethoxysilane; PAMAM—polyamidoamine dendrimer; PANi—polyaniline; CNC—Crystalline nanocellulose; NPAu—gold nanoparticle; *Pt* NC—nano-cluster; GLU—glutaraldehyde; NR—nanorods; KMWNTs—potassium-doped multi-walled carbon nanotubes; PVP—polyvinylpyrrolidone; PPy—polypyrrole; PEDOT—poly-3,4-ethylenedioxythiophene; GCE—glassy carbon electrode.

**Table 3:** Detection of CHOL level (mM) in human serum.

Biosensor Method		Enzymatic Method
ChOx/nCuFe/nPt/GCE	ChOx/nCuFe/GCE	
2.25 ± 0.35	2.23 ± 0.15	2.3 ± 0.2

## Data Availability

The data are included within the present article.

## Supplementary Materials

The following supporting information can be downloaded at https://www.mdpi.com/article/10.3390/jfb14060315/s1, Figure S1: Morphological characteristics of the synthesized NZs nCuFe (a, b) and nCeAu (c, d): SEM images (a, c) and XRM data (b, d); Figure S2. Graphical standard addition method for CHOL assay in the human serum by using sensors ChOx/nCuFe/nPt/GCE (a) and ChOx/nCuFe/GCE; (b) Table S1: HRP-like activities in the solution of the synthesized NZs; Table S2: Analytical characteristics of bionanosensors of architecture ChOx/NZ/GCE (electrode square of 7.06 mm^2^).
