# Supplementary material for: The Peroxidase-like Nanocomposites as Hydrogen Peroxide-Sensitive Elements in Cholesterol Oxidase-Based Biosensors for Cholesterol Assay
Source: J Funct Biomater. 2023 Jun 7;14(6):315. doi: 10.3390/jfb14060315 (PMC10299448; doi:10.3390/jfb14060315)
Supplement: Supplementary file 1 [file jfb-14-00315-s001.zip › jfb-2409679-supplementary.pdf]

Journal of *Functional Biomaterials*

Article

The Peroxidase-like Nanocomposites as Hydrogen Peroxide-Sensitive Elements in Cholesterol Oxidase-Based Biosensors for Cholesterol Assay

Olha Demkiv [1,2], Wojciech Nogala [3], Nataliya Stasyuk [1], Nadiya Grynchyshyn [2], Bohdan Vus [4] and Mykhailo Gonchar [1,*]

1 Institute of Cell Biology, National Academy of Sciences of Ukraine, 79005 Lviv, Ukraine; olgademkiv81@gmail.com (O.D.); stasuk_natalia@ukr.net (N.S.)
2 Faculty of Veterinary Hygiene, Ecology and Law, Stepan Gzhytskyi National University of Veterinary Medicine and Biotechnologies, 79000 Lviv, Ukraine; ngrynchyshyn@ukr.net
3 Institute of Physical Chemistry, Polish Academy of Sciences, 01-224 Warsaw, Poland; wnogala@ichf.edu.pl
4 Department of Electronics and Information Technology, Lviv Polytechnic National University, 79000 Lviv, Ukraine; bogdan.vus@googlemail.com
* Correspondence: mykhailo1952@gmail.com; Tel.: +380-322612144

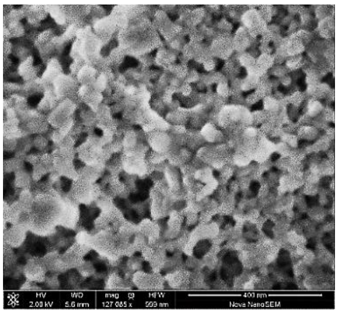

(**a**)

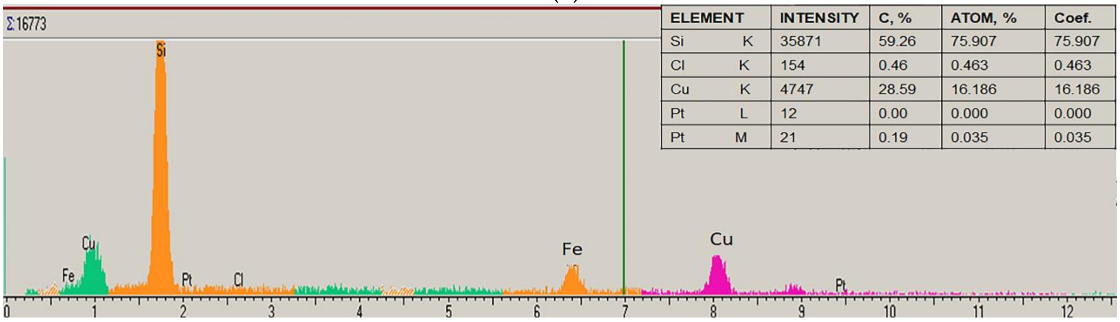

ELEMENT		INTENSITY	C, %	ATOM, %	Coef.
Si	K	35871	59.26	75.907	75.907
Cl	K	154	0.46	0.463	0.463
Cu	K	4747	28.59	16.186	16.186
Pt	L	12	0.00	0.000	0.000
Pt	M	21	0.19	0.035	0.035

(**b**)

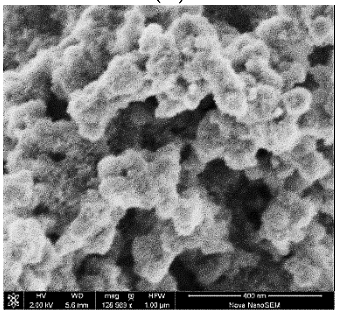

(**c**)

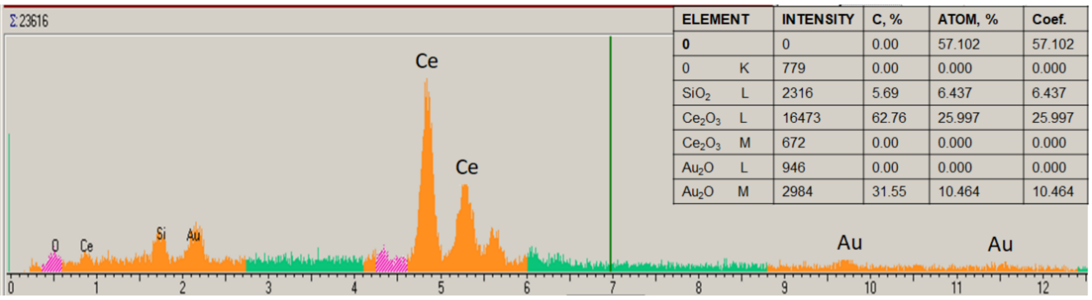

(**d**)

Figure S1. Morphological characteristics of the synthesized NZs nCuFe (**a**,**b**) and nCeAu (**c**,**d**): SEM images (**a**,**c**) and XRM data (**b**,**d**).

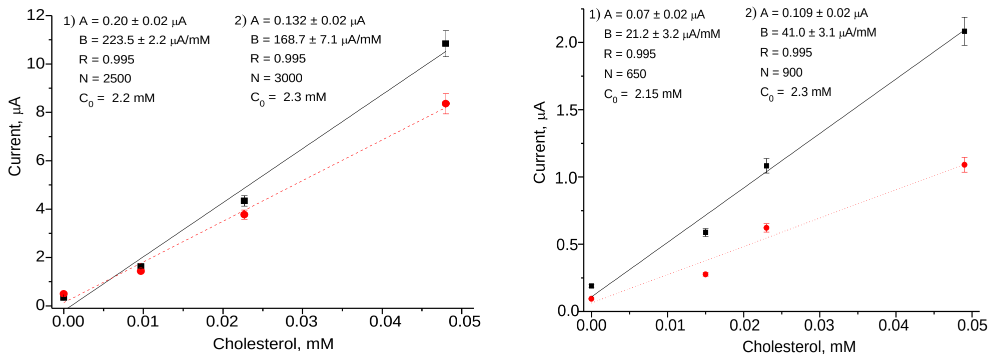

(**a**) (**b**)

Figure S2. Graphical standard addition method for CHOL assay in the human serum by using sensors ChOx/nCuFe/nPt/GCE (**a**) and ChOx/nCuFe/GCE (**b**).

Table S1. HRP-like activities in solution of the synthesized NZs.

N	NZs	Specific Activity, U/mg
1	AuHCF	1.86 ± 0.10
2	CuFe	1.27 ± 0.06
3	CeAu	0.80 ± 0.04
4	CoHCF	0.42 ± 0.02
5	AgCu	0.65 ± 0.03
6	CuPt	0.24 ± 0.01

Table S2. Analytical characteristics of bionanosensors of architecture ChOx/NZ/GCE (electrode square of 7.06 mm^2).

Architecture of the Bioelectrode	I_{max}, nA	Linear Range, up to, mM	K_M^{app}, mM	Sensitivity, $A \cdot M^{-1} \cdot m^{-2}$
GlOx/nCeAu/GCE	33	0.05	1.1	21 ± 0.8
GlOx/nAuHCF/GCE	63	0.16	0.30	30 ± 1.0
GlOx/nCuFe/GCE	321	0.1	0.85	331 ± 4.2
GlOx/HRP/GCE	49	0.12	1.60	49 ± 2.0
